# The relationship between daily positive future thinking and past-week suicidal ideation in youth: An experience sampling study

**DOI:** 10.3389/fpsyt.2022.915007

**Published:** 2022-09-29

**Authors:** Olivia J. Kirtley, Ginette Lafit, Thomas Vaessen, Jeroen Decoster, Catherine Derom, Sinan Gülöksüz, Marc De Hert, Nele Jacobs, Claudia Menne-Lothmann, Bart P. F. Rutten, Evert Thiery, Jim van Os, Ruud van Winkel, Marieke Wichers, Inez Myin-Germeys

**Affiliations:** ^1^Department of Neurosciences, Center for Contextual Psychiatry, Katholieke Universiteit Leuven, Leuven, Belgium; ^2^Leuven Brain Institute, Katholieke Universiteit Leuven, Leuven, Belgium; ^3^Leuven Child and Youth Institute, Katholieke Universiteit Leuven, Leuven, Belgium; ^4^Research Group of Quantitative Psychology and Individual Differences, Faculty of Psychology, Katholieke Universiteit Leuven, Leuven, Belgium; ^5^Department of Psychology, Health, and Technology, Center for eHealth and Well-being Research, University of Twente, Enschede, Netherlands; ^6^Psychiatric Care Sint-Kamillus, Bierbeek, Belgium; ^7^Department of Human Genetics, University Hospital Gasthuisberg, Katholieke Universiteit Leuven, Leuven, Belgium; ^8^Department of Obstetrics and Gynaecology, Ghent University Hospital, Ghent, Belgium; ^9^Department of Psychiatry and Neuropsychology, School for Mental Health and Neuroscience, Maastricht University Medical Center, Maastricht, Netherlands; ^10^Department of Psychiatry, Yale University School of Medicine, New Haven, CT, United States; ^11^University Psychiatric Centre, Katholieke Universiteit Leuven, Kortenberg, Belgium; ^12^Department of Neurosciences, Centre for Clinical Psychiatry, Katholieke Universiteit Leuven, Leuven, Belgium; ^13^Antwerp Health Law and Ethics Chair, University of Antwerp, Antwerp, Belgium; ^14^Department of Psychiatry and Neuropsychology, School for Mental Health and Neuroscience, South Limburg Mental Health and Teaching Network, EURON, Maastricht University, Maastricht, Netherlands; ^15^Faculty of Psychology, Open University of the Netherlands, Heerlen, Netherlands; ^16^Department of Neurology, Ghent University Hospital, Ghent, Belgium; ^17^Brain Center Rudolf Magnus, University Medical Centre Utrecht, Utrecht, Netherlands; ^18^Department of Psychosis Studies, Institute of Psychiatry, King’s Health Partners, King’s College London, London, United Kingdom; ^19^Interdisciplinary Center Psychopathology and Emotion Regulation, University of Groningen, University Medical Center Groningen, Groningen, Netherlands

**Keywords:** suicidal ideation, future thinking, experience sampling method, youth, general population

## Abstract

Reduced positive future thinking has been associated with suicidal ideation and behavior in adults, and appears to be exacerbated by negative affect. Yet, this has received little attention in youth. Prior research has also focused on longer-term future thinking, e.g., months and years, and relied on lab-based assessments. Using the experience sampling method (ESM), we investigated whether short-term future thinking in daily life was associated with suicidal ideation in youth and explored the role of affect in the future thinking–suicidal ideation relationship. A community sample of *N* = 722 adolescent twins and their non-twin siblings completed ESM as part of the TwinssCan study (*n* = 55 with, and *n* = 667 without, past-week suicidal ideation). Participants completed self-report questionnaires, including on past-week suicidal ideation as part of the SCL-90. Subsequently, daily future thinking was assessed each morning for six days with ESM. To investigate the relationship between daily positive future thinking and past-week suicidal ideation, we estimated a mixed-effects linear regression model with a random intercept for participant, including age and sex as covariates. The relationship between daily positive future thinking, past-week suicidal ideation, and average positive and negative affect from the previous day was investigated by estimating two separate mixed-effects linear regression models (one for negative affect, one for positive affect), with a random intercept for participant, and random slopes for average positive and negative affect. Our results showed that participants reporting higher past-week suicidal ideation also reported significantly less daily positive future thinking during the ESM period, and this association remained significant when controlling for previous-day average positive and negative affect. Higher average positive affect from the previous day was significantly associated with higher positive future thinking. Although average negative affect from the previous day was associated with lower positive future thinking, this association was not statistically significant. Our findings indicate that short-term future thinking relates to suicidal ideation among a non-clinical sample of adolescents. Future research should investigate the directionality of the future thinking–suicidal ideation relationship, in order to investigate whether impaired future thinking may be an early warning signal for escalating suicidal ideation in youth.

## Introduction

Being unable to anticipate positive future experiences has been consistently associated with self-harm ideation and behaviors, as have a range of other future oriented constructs (1–7). Previous research has found that individuals who ideate about or engage in self-harm behaviors, generate fewer *positive*–but not more negative-future thoughts than those without a history of self-harm thoughts or behaviors (8–11). Whilst this association appears to be robust, there are several critical unknowns regarding the association between positive future thinking and suicidal ideation that must be addressed if research on positive future thinking is to be translated into clinical practice.

The vast majority of previous studies in this area have measured positive future thinking using the Future Thinking Task [FTT; (8)], in which individuals are asked to free-generate responses to questions regarding what they are looking forward to in one week (including today), one year, and five to ten years. Whilst future thinking within these time-windows prospectively predicts suicidal ideation in adults (12), the extent to which more short-term future thinking, e.g., the next day, relates to suicidal ideation is unknown. Additionally, findings from two studies have demonstrated that positive future thinking decreases following a negative mood manipulation (13, 14), suggesting that the ability to anticipate positive future experiences may be a dynamic process that fluctuates with changes in negative affect. Other research with community samples of adults (15), adolescents (16), and adults with panic disorder and depression (17) has found associations between positive, but not negative, affect, and positive future thinking. To our knowledge, the relationship between naturally occurring, i.e., non-induced, positive and negative affect and positive future thinking has not been examined in individuals experiencing suicidal ideation. Furthermore, as all previous research on future thinking and suicidal thoughts and behaviors has been conducted in the laboratory, we do not know whether positive future thinking as it occurs naturally, in individuals’ daily lives, relates similarly to suicidal ideation.

The laboratory-and survey-based nature of the majority of research on suicidal ideation and behavior limits our capacity to achieve a truly fine-grained understanding of the psychological factors that relate to suicidal ideation and behaviors. To address this, we need to employ methodologies capable of capturing dynamic psychological processes. The Experience Sampling Method [ESM; (18, 19)]—also referred to as Ecological Momentary Assessment [EMA; (20)]—offers an invaluable opportunity to gain insights into individuals’ everyday lives by allowing the collection of dynamic data on individuals’ behaviors and experiences (19). Use of ESM to investigate suicidal ideation has increased rapidly (21–25), building on seminal work by Nock et al. (26), which used ESM to investigate self-injurious thoughts and behaviors. ESM techniques therefore represent an innovative method of investigating dynamic variations in suicidal ideation and behaviors, as well as the psychosocial and environmental factors associated with these thoughts and behaviors.

Whilst there have been a number of studies investigating the relationship between future thinking, and suicidal thoughts and behaviors in adults (8, 9, 14), there has been only one investigation of this in adolescents (27). Intriguingly and contrary to predictions based on the Integrated Motivational Volitional model of suicide [IMV; (28)], 3-month suicidal ideation was associated with defeat and entrapment when positive future thinking was higher; an effect which seemed to be explained by adolescents reporting higher defeat and entrapment generating more unrealistic positive future thoughts (27). That positive future thinking among suicidal youth has received so little attention is surprising, considering that adolescence is an important developmental period for future thinking abilities (29–31). Further, there is a pressing need to better understand factors associated with suicidal ideation and behavior within this age group. In youth, prevalence estimates for suicidal ideation range from 19.8 to 24% (32), and although not all young people who think about suicide will go on to engage in suicidal behavior, among adolescents who experience suicidal ideation, more than one third go on to make a suicide attempt (33). Given that suicide is the fourth leading cause of death worldwide for young people aged 15–29 (34), identification of targets for early intervention in youth—especially for short-term suicide risk—is of critical importance (35). In a recent review of research on youth suicide, Cha et al. (36) further emphasized the need to devote greater attention to the psychological factors associated with suicidal ideation and behaviors in youth in the short-term. Moreover, they also highlighted the overreliance on traditional self-report methods when investigating factors associated with suicidal ideation and behavior in youth and encouraged researchers to “move beyond traditional tools used in psychology research…” [p473; (36)].

In order to address these open questions and issues regarding the relationship between short-term positive future thinking and suicidal ideation in youth, we conducted a study using pre-existing ESM data to investigate whether daily positive future thinking was associated with past-week history of suicidal ideation in youth. We hypothesized that higher past-week suicidal ideation would be significantly and negatively associated with daily positive future thinking. Based on previous research [e.g., (13, 14)] suggesting that affect influences and, in some cases, potentiates a decrease in positive future thinking, we also conducted exploratory analysis to investigate the relationship between past-week suicidal ideation and daily positive future thinking, with and without positive and negative affect.

## Materials and methods

### Participants and recruitment

Participants were from the TwinssCan study (37), a large general population-based cohort of adolescent twins and their young adult non-twin siblings, which formed part of the East Flanders Prospective Twin Survey [EFPTS; (38)]. Twin participants were aged 15–18 years old at the time of enrollment and their non-twin siblings were aged 15–35 years old. Exclusion criteria were: (1) not understanding the purpose of the study and being unable to provide informed assent/consent; (2) not having parent/caregiver consent (if under 18 years old); (3) presence of pervasive mental disorder; and (4) being unable to complete the study procedure, or providing invalid/unreliable data on measures. The total TwinssCan ESM dataset (version 2.3) sample comprised *N* = 840 individuals; however, *n* = 13 participants had missing data in ESM design variables required for multilevel modeling of the data, so were excluded. For the purposes of this study we specifically focused on youth,^1^ so excluded *n* = 39 participants older than 25, leaving a sample of 788 participants before additional data cleaning (see Data Cleaning section below for further details). No information regarding participants’ ethnicity was available; however, as a proxy, participants were asked whether they spoke any languages other than Dutch at home. A small number of participants indicated yes (*n* = 27), the majority indicated no (*n* = 705), and *n* = 11 participants’ responses were missing. Participants’ perceived social status was assessed using the MacArthur Scale of Subjective Social Status (39, 40), wherein they were shown an illustration of a ten-rung ladder, with each rung corresponding to individuals’ position within the community. Using a 1–100 visual analogue scale, researchers asked participants to indicate their position on the ladder for the community that was most relevant to them. Within the current study, participants’ mean score on the ladder was 37.09 (SD: 27.57), and the median score was 50. The study received local ethics committee approval (Medical Ethics Committee UZ/KU Leuven, No. B32220107766), and informed consent was obtained from all participants (and parents where participants were aged < 18 years old).

### Procedure

The procedure for the TwinssCan study is fully described elsewhere (37). During a baseline interview session with a member of the TwinssCan research team, participants completed a battery of self-report questionnaires, including questions regarding various psychosocial factors and experiences of psychopathology symptoms. At the end of the baseline session, the researcher briefed participants regarding the ESM protocol, where participants were asked to complete a series of brief questionnaires, ten times per day for six days, plus additional shorter morning and evening questionnaires. Future thinking was measured during the morning questionnaire and positive and negative affect were assessed in the momentary questionnaires. All ESM measures were administered using Psy-mate©, a custom-made Personal Digital Assistant (41), which emitted a notification prompting participants to complete a questionnaire. Notifications were given between 7:30 am and 10:30 pm, thus the morning questionnaire was available to participants from 7:30 am.

### Measures

#### Past-week suicidal ideation

Past week suicidal ideation was assessed using a single item from the Dutch version of the SCL-90-R (42) administered at baseline, which asked “[How often in the past week including today have you been troubled by] thoughts of ending your life?” Responses were given on a five-point Likert-type scale ranging from 0 (not at all) to 4 (extremely).

#### Future thinking

To assess daily future thinking, we used the Experience Sampling Method (18, 19). In the current study, future thinking was assessed using a single item from the ESM morning questionnaire, which asked participants to indicate “How much you are looking forward to today” on a seven-point Likert-type scale ranging from 1 (not at all) to 7 (extremely). The intraclass correlation coefficient (ICC), which indicates the amount of variance that is due to between-person differences, was 0.30 for the future thinking ESM item, and the adjusted and conditional ICC values were identical. The morning questionnaire was presented to participants once a day for a period of six days and was the first questionnaire received by participants each day. The morning questionnaire included a total of five items.

#### Previous day mean positive and negative affect

Between the morning and evening questionnaires, 10 momentary questionnaires were administered consisting of a maximum of 57 items (depending upon answers to conditionally branched questions). Following receipt of the prompt, participants had 15 min to respond to questionnaires. During each of the 10 momentary ESM questionnaires, participants were presented with items assessing positive and negative affect. All affect items began with the stem “I feel…” followed by five items assessing positive affect (cheerful, relaxed, satisfied, enthusiastic, and generally well) and four items assessing negative affect (insecure, lonely, anxious, and annoyed). Responses to all items were provided on a seven-point Likert-type scale, ranging from 1 (not at all) to 7 (very much so). Separate means were calculated for positive and negative affect at each beep by averaging item scores. These questionnaire-level means were then aggregated within days to create average daily positive and negative affect scores, which were then lagged to provide average positive and daily negative affect scores for the day prior to completion of the morning questionnaire. ICCs for the mean positive and negative affect variables were 0.58 and 0.65, respectively, and values for adjusted and conditional ICCs were identical. Within-and between-person reliability (ω) for positive affect were 0.82 and 0.92, and 0.78 and 0.91 for negative affect.

### Data cleaning

Participants who completed fewer than 30% of questionnaires during the ESM period were asked to continue with ESM for longer than the 6-day protocol. Compliance with ESM protocols, defined as the proportion of completed questionnaires out of the total number of questionnaires delivered, is related to a number of different participant characteristics, including presence of psychopathology symptoms (43), therefore to minimize heterogeneity within the sample, we excluded participants with ESM compliance below 30%. This resulted in a sample size of *N* = 743. Following concerns regarding use of the 30% compliance rule of thumb as a basis for excluding participants (44), we also conducted an exploratory sensitivity analysis in which we re-estimated all models including participants with compliance below 30%. The results of these sensitivity analyses are presented in Supplementary 1 and were virtually identical to the results of the analyses excluding participants with < 30% compliance.

#### Statistical analysis

##### Open research practices

The research questions, hypotheses, variables, data inclusion/exclusion criteria, and statistical analysis plan were postregistered on the Open Science Framework,^1^ a type of preregistration occurring after data collection, but before data analysis (45). Prior to postregistration, data had not been accessed.

Subsequent to registration and data access, a number of issues emerged and consequently, several major aspects of our preregistered plan were changed. These changes are detailed in a supplement to our original registration^2^ and in more detail in the Supplementary materials (Supplementary 2). Briefly, the major deviation from our original postregistered analysis plan was a change in our independent variable from past-year suicidal ideation and attempts, to past-week suicidal ideation. This was due to unexpected conditional branching within the CIDI questionnaire, which meant that too few individuals had received the questions pertaining to past-year suicidal ideation and behavior for meaningful analysis to be feasible. For transparency, we report the results of these original postregistered analyses in Supplementary 3.

The full ESM questionnaire from the TwinssCan study (37) and R Markdown files for all analyses from the current study are available on the Open Science Framework.^3^ The Supplementary materials for the current manuscript are also available on the OSF project page for this study.

##### Analysis plan

The statistical analysis plan presented here is that outlined in the supplementary registration, not the original registration. Data were cleaned and analyzed using R v4.1.2 (46) *via* R Studio v2022.2.0.443 (47). As observations (level 1) were nested within participants (level 2), mixed effects linear regressions with random intercepts were conducted using the “lmer” function from the “lme4” package (48) for analyses of past week suicidal ideation and daily future thinking. Tables of model summaries were generated with the “SjPlot” package v2.8.10 (49) and plots were generated using “ggeffects” v1.1.1.1 (50) and “cowplot” v1.1.1 (51). Intraclass Correlation Coefficients (ICCs) and (where possible) reliability for ESM items were calculated using the “psych” package (52) v1.5.8 and the “multilevel tools” package (53) v0.1.1. Appropriateness of multilevel modeling for these data was assessed by estimating an unconditional model, which included only the outcome variable (daily future thinking) and the random intercept (participant). As the unconditional model was statistically significant (*p* < 0.001), multilevel modeling was considered appropriate for these data (54, 55). For analyses of the relationship between past week suicidal ideation and daily future thinking including affect covariates, two separate mixed effects linear regressions with random intercepts and random slopes were estimated, one including average positive affect and one including average negative affect, from the previous day. In all analyses, daily future thinking was the dependent variable and past-week suicidal ideation was the independent variable. The past-week suicidal ideation variable was grand mean-centered, i.e., centered using the mean calculated across all participants, in order to aid interpretability of the intercept (56). Average lagged positive and negative affect were participant mean-centered. This is recommended for time-varying variables in order to avoid conflation between the association between the dependent variable and variation of the independent variables at the within-and between-person level (56). Given associations between sex, age, and both suicidal ideation (57, 58) and future thinking (31), we included both age and sex as covariates. The covariance structure of the Level 1 within-person errors was assumed to be independent. As stated in our original post-registration, due to the absence of comparable published literature from which to draw parameters for simulation-based power calculations, we did not conduct an *a priori* or sensitivity power calculation.

## Results

**FIGURE 1:**
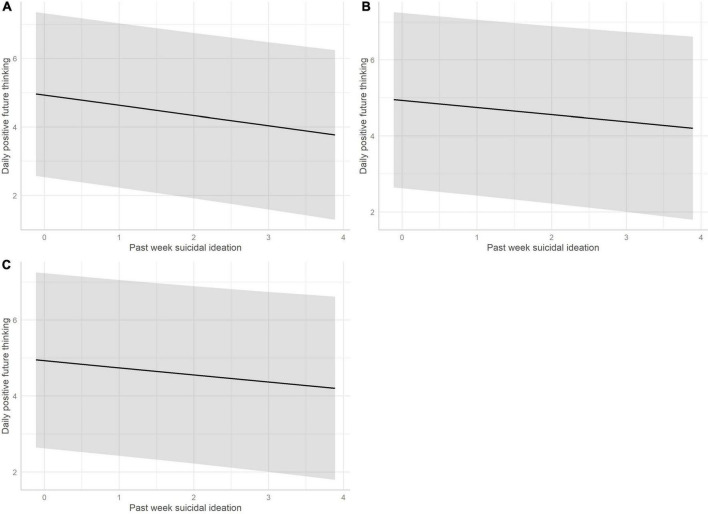
The association between daily positive future thinking and past-week suicidal ideation, controlling for (A) age and sex; (B) age, sex, and average positive affect from the previous day; and (C) age, sex, and average negative affect from the previous day. Shading represents 95% confidence interval.

Fifty-five (7.4%) participants reported experiencing suicidal ideation within the past week, relative to 677 (89.8%) participants who reported no past-week suicidal ideation. Twenty-one (2.8%) participants were missing responses to the suicidal ideation item. Mean compliance, i.e., mean number of completed ESM questionnaires out of the total 60, within the full sample was 41.72 (SD: 10.49; 69.53% compliance). Neither age (β = 0.00958, *SE* = 0.015, *p* = 0.53) nor sex (β = 0.00149, *SE* = 0.075, *p* = 0.98) were significantly related to positive future thinking. However, both were related to past-week suicidal ideation: younger age was associated with higher suicidal ideation (β = −0.016, *SE* = 0.003, *p* < 0.001) and youth reporting suicidal ideation were more likely to be female (β = 0.046, *SE* = 0.014, *p* = 0.00075). For sample and variable descriptive information, see Tables 1, 2 for variable descriptives according to endorsement vs. non-endorsement of past-week suicidal ideation. Results of the analyses investigating the relationship between past-week suicidal ideation and daily future thinking (with and without affect) are reported below. Results of multilevel analyses are presented in Figure 1 and Table 3.

**TABLE 1:** Sample and variable descriptives.

	Mean (SD)	Median	Range
Age (years)	16.85 (2.39)	16.00	15–25
Sex (% female)	58.5%	–	–
Past week suicidal ideation	0.108 (0.44)	0	0–4
Daily positive future thinking	4.91 (1.04)	5	1–7
Average daily positive affect	5.01 (0.68)	5.07	2.19–6.77
Average daily negative affect	1.78 (0.56)	1.68	1.01–4.51

**TABLE 2:** Variable descriptives for adolescents reporting vs. not reporting past-week suicidal ideation.

	Past-week suicidal ideation			No past-week suicidal ideation		
		
	Mean (SD)	Median	Range	Mean (SD)	Median	Range
Past week suicidal ideation	1.42 (0.85)	1	1–4	–	–	–
Daily positive future thinking	4.52 (1.14)	4.5	1–6.6	4.94 (1.022)	5	1–7
Average daily positive affect	4.53 (0.94)	4.58	2.19–6.55	5.05 (0.64)	5.09	3.17–6.77
Average daily negative affect	2.25 (0.79)	2.04	1.24–3.76	1.74 (0.51)	1.66	1.007–3.81

**TABLE 3:** Multilevel model summaries.

	Daily positive			Daily positive			Daily positive			Daily positive		
				
	future thinking			future thinking			future thinking			future thinking		
				
Predictors	Estimates	CI	*p*	Estimates	CI	*p*	Estimates	CI	*p*	Estimates	CI	*p*
(Intercept)	4.93	4.86 to 5.00	**< 0.001**	4.82	4.28 to 5.36	**< 0.001**	4.77	4.21 to 5.34	**< 0.001**	4.75	4.19 to 5.31	**< 0.001**
Past week suicidal ideation				−0.30	−0.46 to −0.13	**< 0.001**	−0.19	−0.36 to −0.01	**0.041**	−0.19	−0.37 to −0.02	**0.031**
Age				0.01	−0.02 to 0.04	0.741	0.00	−0.03 to 0.03	0.872	0.00	−0.03 to 0.03	0.814
Sex				0.02	−0.13 to 0.16	0.838	0.07	−0.08 to 0.22	0.368	0.07	−0.08 to 0.22	0.377
Average positive affect from previous day							0.23	0.11 to 0.36	**< 0.001**			
Average negative affect from previous day										−0.11	−0.29 to 0.07	0.236
**Random effects**												
σ^2^	1.50			1.49			1.38			1.37		
τ_00_	0.64_subjid_			0.62_subjid_			0.62_subjid_			0.62_subjid_		
τ_11_							0.34_subjid.cent_day_pa_lag_			0.87_subjid.cent_day_na_lag_		
ρ_01_							−0.10_subjid_			−0.16_subjid_		
ICC	0.30			0.30			0.33			0.34		
N	737_subjid_			716_subjid_			709_subjid_			708_subjid_		
Observations	3474			3377			2622			2617		
Marginal R^2^/conditional R^2^	0.000/0.300			0.008/0.301			0.009/0.338			0.004/0.343		
AIC	12079.709			11723.155			9099.862			9090.215		

σ^2^ = Within-group residual variance; τ_00_ = Between-group variance; τ_11_ = Random-slope variance; ρ_01_ = Random-slope intercept correlation.

There was a significant negative association between past-week suicidal ideation and daily positive future thinking, such that youth who reported higher levels of suicidal ideation within the past-week also reported lower levels of daily positive future thinking during the ESM period. Higher levels of daily positive future thinking were associated with reporting higher average positive affect on the preceding day. Higher levels of daily positive future thinking were associated with reporting lower average levels of negative affect the previous day (i.e., a negative association), however this association was not statistically significant and should therefore be interpreted with caution.

## Discussion

In the current study, we found that past-week suicidal ideation was significantly associated with daily positive future thinking in a non-clinical, youth sample. Higher levels of daily positive future thinking were associated with lower levels of past-week suicidal ideation. Both average negative and positive affect from the previous day were associated with daily positive future thinking, such that youth experiencing higher positive and lower negative affect during the previous day also experienced higher levels of positive future thinking the next day. However, only the association between previous day positive affect and daily positive future thinking was statistically significant. The non-significant negative association between previous day negative affect and daily positive future thinking should therefore be interpreted with caution.

Our results are consistent with previous laboratory-based studies, finding that individuals who have thought about or engaged in self-harm also exhibit reduced positive future thinking (8–11). All previous research, however, has focused on positive future thinking over longer time-frames, generally including the next week, month, year, and five to ten years, and our study provides the first evidence indicating that short-term positive future thinking is also associated with recent suicidal ideation. Moreover, we show that future thinking in youths’ normal, everyday lives–outside of the controlled laboratory environment–relates to recent thoughts of suicide. Previous research using ESM has found that relationships between suicidal ideation and other risk factors, previously established with self-report questionnaires, do not always translate to everyday life. For example, despite a large body of questionnaire-based research associating perceived burdensomeness and thwarted belongingness with suicidal ideation [e.g., (59, 60)], ESM research has not replicated these associations (24). This suggests that some risk and protective factors are less dynamic, and associated with suicidal ideation over longer timeframes, e.g., weeks, months, or years, whereas others are more dynamic and relate to suicidal ideation over minutes, hours, or days. Such dynamic factors may also be more amenable to change. That both short-term and longer-term positive future thinking both appear to be related to suicidal ideation may indicate the temporal robustness of future thinking as a correlate of suicidal ideation.

Combined with results of previous research with clinical populations [e.g., (10, 11, 14)], our finding that daily positive future thinking relates to even very low levels of recent suicidal ideation in a non-clinical youth sample, suggests that impaired positive future thinking could be present even at the very early stages of suicidal ideation. The need to examine risk factors for suicidal ideation in non-clinical samples has recently been highlighted by Millner et al. (61), who argue that risk factors for the development of suicidal ideation can likely not be derived from research with samples who have already experienced severe ideation or engaged in suicide attempts. Processes involved in the genesis of suicidal thoughts must necessarily also be present among individuals experiencing no or low levels of suicidal ideation, in order for changes in these processes to cause escalation in suicidal ideation (61). In this regard, future thinking may hold promise, as it appears to be a process associated with suicidal ideation across the spectrum of severity.

In the current study, the relationship between daily positive future thinking and past-week suicidal ideation remained significant even when controlling for previous-day positive and negative affect. This suggests that past-week suicidal ideation is associated with variance in daily future thinking beyond the effects of average positive and negative affect from the previous day. We also found a significant positive association between daily positive future thinking and positive affect from the previous day. This is consistent with previous studies demonstrating an association between positive affect and positive future thinking (15–17). Although we found a negative association between negative affect from the previous day and positive future thinking, this association was not statistically significant. Early studies of future thinking (15–17) found no association between negative affect and positive future thinking. Yet, these earlier studies’ findings also appear at odds with later research demonstrating that negative mood inductions reduced positive future thinking (13, 14). There is evidently heterogeneity within the literature regarding the relationship between affect and positive future thinking, which warrants further exploration.

Although previous literature indicates that adolescence is a sensitive period for the development of future orientation abilities (29–31), we found no significant association between age and daily positive future thinking. The age-range of our sample was 15–25 years old, covering periods of developmental flux in future thinking identified in prior studies (31), thus our study should have been able to capture developmental differences in future thinking, had they been present. One explanation for the lack of association between age and future thinking observed in the current study is that developmental differences may be more apparent in static, as opposed to dynamic, measures of future thinking. As our study is, to the best of our knowledge, the first to investigate dynamic daily life future thinking, this hypothesis should be interrogated further in future research. It may also be possible that age-related differences in future thinking may have been apparent in a younger sample (e.g., 12–14 year-olds). Future research should also investigate future thinking in the daily lives of younger youth.

### Strengths and limitations

Our study has a number of strengths. To our knowledge, this is the first investigation of positive future thinking in daily life and its association with recent suicidal ideation among a non-clinical sample of adolescents. Use of ESM to assess positive future thinking increases ecological validity (19), sheds new light on temporal aspects of future thinking, and provides the first indication that naturally occurring, positive future thinking relates to suicidal ideation. These findings therefore have theoretical and practical relevance for suicide research, where future thinking features within the IMV model of suicide (28). Only one previous study has investigated the relationship between suicidal ideation and future thinking in adolescents (27), thus our study adds to and extends the literature on this topic, as well as the broader future thinking and suicide, and developmental future orientation, literature.

The field of suicide research, as well as clinical psychology and psychiatry more broadly, have been highlighted as in need of a greater focus on transparency, reproducibility, and replicability (62–66). To this end, we have made our analysis code available on the Open Science Framework (open code). We also postregistered our hypotheses and analysis plan. However, due to unexpected conditional branching in the dataset and consequently low numbers of individuals reporting suicidal ideation in our original independent variable, significant changes to our post-registration were necessary. We made every effort to document these deviations as transparently as possible, with a supplementary registration and a full description of deviations in the Supplementary materials. However, this undeniably compromised several key goals of preregistration (67–69) and we fully appreciate this is a limitation of our study.

We must also acknowledge several further limitations. The number of adolescents endorsing past-week suicidal ideation was low (*n* = 55) in this general population adolescent sample and even among those reporting past-week suicidal ideation, the mean level of suicidal ideation was low (1.43 on a 1–4 scale). Our findings therefore require further replication in larger samples, both with adolescents from the general population, as well as individuals endorsing higher levels of suicidal ideation. The difference in mean levels of daily positive future thinking between those with vs. without past-week suicidal ideation was also small. The extent to which daily positive future thinking may be predictive of clinically relevant suicidal ideation outcomes cannot be determined from this study, and should be tested in future prospective research with clinical samples, using validated suicidal ideation scales. However, an additional challenge for determining the clinical meaningfulness of effects is that effects cannot be compared easily across ESM studies due to difficulties in obtaining standardized effect size estimates (70).

Additionally, we assessed suicidal ideation using a single-item from the SCL-90 (42) and previous research has highlighted single-item assessments of suicidal ideation and behavior as suboptimal (71). As our study used pre-existing data from the TwinssCan study (37), we were constrained by the variables available within the dataset. Future thinking was also assessed using only a single item, which naturally limits the scope and level of future thinking we could capture. However, given the intensive nature of ESM data collection, use of single-item measures to assess constructs of interest is common (72). The single item we used to assess future thinking in the current study has not been psychometrically validated and as—to the best of our knowledge—this is the first study of future thinking in daily life, the extent to which this item demonstrates convergent validity with other measures of future thinking is unknown. There are currently no standard items for assessing future thinking (or related constructs) using ESM. Indeed, ESM suffers from a general lack of validated questions for assessing constructs (72–74). Although initiatives such as the ESM Item Repository (75) are underway to build sets of psychometrically valid ESM items, future research should also invest in basic measurement groundwork to develop high quality items for assessing future thinking in daily life. Other laboratory research has underscored the relevance of assessing the content of positive future thoughts (10) and the likelihood of positive future events occurring (27), in relation to suicidal ideation. When developing ESM measures to assess future thinking, content and likelihood of future thoughts should also be considered.

Positive and negative affect were not assessed at the same moment as future thinking, because future thinking was included only in the morning questionnaire and affect only in the momentary questionnaires. We used average affect from the previous day, as this was temporally closest to completion of the morning questionnaire. However, this may have been too long a time-window to detect meaningful dynamic effects of affect upon future thinking. Subsequent research should investigate the relationship between affect and future thinking contemporaneously, as well as prospectively from one moment to the next.

As post-registered, we did not conduct a power calculation, because the lack of comparable literature and available data meant we had nothing from which to draw meaningful parameters for a power calculation. Power and sample size calculations are often neglected in ESM research (64, 76, 77) and future research should aim to replicate our findings in larger samples, guided by simulation-based power analyses.

Finally, although not specifically a limitation, it is worth noting that data are drawn from a sample of twins and their non-twin siblings. There is some debate regarding whether twins are representative of the general population, termed the “twin representativeness assumption” (78). However, researchers have argued that although some differences are apparent—for example, in internalizing symptoms and eating disorders—the small to moderate effect sizes of these differences suggest that results from twins can be generalized to non-twins (79). Nevertheless, the potential effect of twinness on positive future thinking is an empirical question and future research should investigate this using dyadic models that are able to account for the interdependence between participants in twin pairs [e.g., (80)]. We also suggest that the findings of the current study should be replicated in non-twin samples.

## Future research and clinical directions

Our findings provide several avenues for further research. First, future thinking has been linked to suicidal ideation and behavior among individuals with chronic pain (81), highlighting another group that could also benefit from further investigation of the relationship between short-term, positive future thinking and suicidal ideation. Second, future research should investigate future thinking in conjunction with other risk and protective factors for suicidal ideation. In the current study, we examined positive future thinking in isolation, but recent research (27, 82) and contemporary theoretical models of suicide—the IMV (28)—highlight that future thinking may influence suicidal ideation in concert with other factors. For example, within the IMV model, future thinking is posited as a “motivational moderator,” disrupting or facilitating the pathway between defeat, entrapment, and suicidal ideation (28). Third, the relationship between future thinking, suicidal ideation and affect may feasibly differ as a function of context. Research should assess future thinking and suicidal ideation at the momentary level, to determine whether the future thinking—suicidal ideation association is robust across contexts. Future studies could also substantively examine the role of context in positive future thinking, to reveal whether particular contexts, e.g., being in company, are associated with more positive future thinking. Fourth, and finally, the ICCs observed in the current study suggest that meaningful variance can also be explained at the within-as well as the between-person level. Further research would benefit from examining potential within-person relationships between future thinking and suicidal ideation.

Clinically, given the lack of knowledge regarding short-term correlates of suicidal ideation and behavior (83, 84), we consider these to be promising findings which, following further replication, may provide novel targets for rapid interventions to prevent suicidal thoughts and behaviors in young people, especially from non-clinical populations. Ecological Momentary Interventions (85), including Just-in-time adaptive interventions (86), could be an optimal approach to target positive future thinking in youths’ daily lives. These could also be blended with existing, e.g., Future Oriented Group Training (87) and emerging, e.g., “Edge of the Present” virtual reality therapy (88), interventions for future thinking.

## Conclusion

The current study provides the first evidence to suggest that short-term (daily) positive future thinking is associated with past-week suicidal ideation in a non-clinical sample of adolescents. These findings are consistent with the broader experimental literature on future thinking and its association with suicidal ideation and behavior. Whilst the general population nature of the sample resulted in a relatively low number of adolescents endorsing recent suicidal ideation, our study indicates that daily positive future thinking is a promising avenue for future research.

## Data availability statement

The data analyzed in this study is subject to the following licenses/restrictions: Data are not publicly available and are available upon application to the TwinssCan research team. Requests to access these data should be directed to TwinssCan project team: info@twinsscan.eu.

## Ethics statement

The studies involving human participants were reviewed and approved by Medical Ethics Committee UZ/KU Leuven, No. B32220107766. Written informed consent to participate in this study was provided by all participants or participants’ legal guardian/next of kin when participants were under 18 years old.

## Author contributions

OJK and IM-G: conceptualization. SG: data curation. OJK: formal analysis, investigation, visualization, and writing – original draft. OJK, GL, TV, and IM-G: methodology. GL: software. OJK, GL, TV, JD, CD, SG, MD, NJ, CM-L, BR, ET, JO, RW, and IM-G: writing – review and editing. All authors contributed to the article and approved the submitted version.

## Footnotes

1
https://osf.io/6mja2

2
https://osf.io/4nck7

3
https://osf.io/up63x/
