# Supplementary material for: The relationship between daily positive future thinking and past-week suicidal ideation in youth: An experience sampling study
Source: Front Psychiatry. 2022 Sep 29;13:915007. doi: 10.3389/fpsyt.2022.915007 (PMC9556869; doi:10.3389/fpsyt.2022.915007)
Supplement: Supplementary file 1 [file Data_Sheet_1.PDF]

Supplement 1: Sensitivity analysis

The relationship between daily positive future thinking and past-week suicidal ideation in youth: An experience sampling study

Olivia J. Kirtley, Ginette Lafit, Thomas Vaessen, Jeroen Decoster, Catherine Derom, Sinan I. Gülöksüz, Marc De Hert, Nele Jacobs, Claudia Menne-Lothmann, Bart P. F. Rutten, Evert Thiery, Jim van Os, Ruud van Winkel, Marieke Wichers, Inez Myin-Germeys

In the main analyses presented in the manuscript, we applied an exclusion criteria based on compliance, such that only participants who completed 30% or more of the ESM questionnaires were included within our analyses. Although this is a common rule of thumb within ESM research, compliance-based exclusion criteria have recently been questioned (Jacobson, 2019). With this in mind, here we present a sensitivity analysis in which we conduct our main analyses again including participants with lower than 30% ESM compliance. As these analyses were *post-hoc*, they should be regarded as exploratory.

Sample description:

Including participants with less than 30% compliance, the total sample comprises N=788 individuals, of whom n=706 report no past-week suicidal ideation and n=57 report past-week suicidal ideation (n=25 participants had missing data for this variable). The majority (58.12%) of the sample was female. Mean age within the sample was 16.84 years old (SD: 2.38), with a range of 15 – 25 years old. Mean score on the 'social ladder' within this sample was 37.48 (SD: 27.54). N=747 participants reported speaking only Dutch at home, whereas n=27 reported speaking a language other than Dutch at home.

Results

In the full sample, the average daily positive future thinking score was 4.912 (SD: 1.05) and the mean past-week SI score was .108 (SD: .44). Average daily negative affect from the previous day was 1.78 (SD: .55), and average daily positive affect from the previous day was 5.02 (SD: .69). Neither sex (β = .035, SE = .073, p = .63) nor age (β = .012, SE = .015, p = .49) were significantly associated with daily positive future thinking. However both age (β = -.016, SE = .0027, p <.001) and sex (β = .034, SE = .013, p = .0098) were significantly associated with past-week suicidal ideation, such that individuals reporting higher past-week suicidal ideation were younger and more likely to be female.

Main analyses

The results of the main analyses in this sample can be found in Table S1 below.

There was a significant negative association between daily positive future thinking and past-week suicidal ideation, such that higher levels of past-week suicidal ideation were associated with lower levels of daily positive future thinking,

Table S1
Multilevel model summaries for sensitivity analysis including participants with <30% ESM compliance

	**Daily positive future thinking**			**Daily positive future thinking**			**Daily positive future thinking**			**Daily positive future thinking**		
Predictors	*Estimates*	*CI*	*p*	*Estimates*	*CI*	*p*	*Estimates*	*CI*	*p*	*Estimates*	*CI*	*p*
(Intercept)	4.92	4.85 – 4.99	**<0.001**	4.79	4.26 – 5.33	**<0.001**	4.74	4.19 – 5.29	**<0.001**	4.72	4.17 – 5.27	**<0.001**
Past week suicidal ideation				-0.29	-0.46 – -0.13	**<0.001**	-0.18	-0.36 – -0.01	**0.040**	-0.19	-0.37 – -0.02	**0.031**
Age				0.00	-0.02 – 0.03	0.766	0.00	-0.03 – 0.03	0.873	0.00	-0.03 – 0.03	0.806
Sex				0.04	-0.11 – 0.18	0.611	0.09	-0.06 – 0.24	0.227	0.09	-0.06 – 0.24	0.226
Average positive affect from previous day							0.22	0.10 – 0.34	**<0.001**			
Average negative affect from previous day										-0.09	-0.27 – 0.08	0.286
**Random Effects**												
σ^2	1.48			1.47			1.37			1.36		
τ_{00}	0.65 $_{subjid}$			0.62 $_{subjid}$			0.61 $_{subjid}$			0.61 $_{subjid}$		
τ_{11}							0.32 $_{subjid.cent\_day\_pa\_lag}$			0.81 $_{subjid.cent\_day\_na\_lag}$		
ρ_{01}							-0.11 $_{subjid}$			-0.13 $_{subjid}$		
ICC	0.30			0.30			0.33			0.34		
N	778 $_{subjid}$			754 $_{subjid}$			745 $_{subjid}$			744 $_{subjid}$		
Observations	3591			3487			2706			2702		
Marginal R^2 / Conditional R^2	0.000 / 0.303			0.008 / 0.302			0.009 / 0.338			0.005 / 0.342		
AIC	12455.122			12067.326			9361.204			9354.539		

Average positive affect from the previous day was significantly associated with daily positive future thinking, such that higher positive affect was associated with more daily positive future thinking. Although there was a negative association between average negative affect from the previous day and daily positive future thinking, this association was not statistically significant.
