# Supplementary material for: The relationship between daily positive future thinking and past-week suicidal ideation in youth: An experience sampling study
Source: Front Psychiatry. 2022 Sep 29;13:915007. doi: 10.3389/fpsyt.2022.915007 (PMC9556869; doi:10.3389/fpsyt.2022.915007)
Supplement: Supplementary file 2 [file Data_Sheet_2.PDF]

Supplement 2: Deviations from postregistration

The relationship between daily positive future thinking and past-week suicidal ideation in youth: An experience sampling study

Olivia J. Kirtley, Ginette Lafit, Thomas Vaessen, Jeroen Decoster, Catherine Derom, Sinan I. Gülöksüz, Marc De Hert, Nele Jacobs, Claudia Menne-Lothmann, Bart P. F. Rutten, Evert Thiery, Jim van Os, Ruud van Winkel, Marieke Wichers, Inez Myin-Germeys

There were several major deviations from our original postregistered analysis plan (see: https://osf.io/6mja2) due to some unforeseen conditional branching in the dataset. Subsequently, we made a supplementary postregistration (see: https://osf.io/4nck7), following data access but prior to data analysis. In the current supplement, we provide a more detailed description of all deviations from the original postregistration.

First, after data had been accessed, it became evident that the presentation of the item regarding past year suicidal ideation in the CIDI was dependent upon the answer to three stem questions asking whether in the last two weeks participants had: 1) felt sad, depressed or empty; 2) lost interest in most things like work, hobbies, and other things they usually enjoy; 3) felt irritable or grouchy or in a bad mood most of the time. Therefore, only individuals who answered "yes" to one of these three questions received the item on past year suicidal ideation (and consequently the items on suicidal plans and attempts). This resulted in an extremely small sample size for past year suicidal ideation (N=18), but also meant that depressive symptoms could not be included as a covariate for this analysis, as it was a selection criterion for item administration. Participants reporting who received the question on past year suicidal ideation were then regarded as a subgroup with depressive symptoms. For transparency and consistency with our original pre-registration, we still report the results from these analyses, however, given the sample size and likely low inferential value of these results, we include them only in the supplementary materials (**Supplement 2**).

Second, of those who received the CIDI item regarding suicidal ideation, only three reported having made a suicide attempt within the last year. This unfortunately precluded any meaningful analysis of suicide plans or behaviours.

Third, as a result of the above issues, we changed our hypotheses to focus only on suicidal ideation in the past year (assessed via the CIDI item). As this analysis could only be conducted in a greatly reduced sample size, who were "selected" based on endorsement of depressive symptoms, we changed our main analysis to use past-week suicidal ideation as the predictor variable. Past-week suicidal ideation was assessed in the SCL-90-R using a single item "How often in the past week including today have you been troubled by thoughts of ending your life?" and this item was

administered to the full sample. The analysis including past-week suicidal ideation as the independent variable are reported in the manuscript as the main analyses. The original postregistered analyses including past-year suicidal ideation as the independent variable are reported in **Supplement 2**.

Fourth, in the original registration, we planned to calculate average combined positive and negative affect across the whole ESM week and include this as a covariate in our analyses. Subsequently, we discussed this further within the team following reconsideration of additional literature used during preparation of another project. This suggested our original approach may have underestimated the potential role of temporality of negative affect in the relationship between future thinking and suicidal ideation. As neither positive nor negative affect was assessed during the morning questionnaire, i.e. contemporaneously with future thinking, we therefore decided to calculate separate lagged variables for average positive and negative affect from the previous day. Separate models were estimated including lagged positive and negative affect. We also estimated models excluding affect covariates.

Fifth, we conducted an exploratory sensitivity analysis to investigate whether results differed depending on inclusion/exclusion of individuals with <30% compliance on the ESM questionnaires. We decided to investigate this based on discussions arising within the field about the potential disadvantages of using this rule of thumb for compliance-based exclusion (Jacobson, 2019). The results of this sensitivity analysis are reported in **Supplement 1**.
