# Supplementary material for: The relationship between daily positive future thinking and past-week suicidal ideation in youth: An experience sampling study
Source: Front Psychiatry. 2022 Sep 29;13:915007. doi: 10.3389/fpsyt.2022.915007 (PMC9556869; doi:10.3389/fpsyt.2022.915007)
Supplement: Supplementary file 3 [file Data_Sheet_3.PDF]

# Supplement 3: Planned analyses from original post-registration

# The relationship between daily positive future thinking and past-week suicidal ideation in youth: An experience sampling study

Olivia J. Kirtley, Ginette Lafit, Thomas Vaessen, Jeroen Decoster, Catherine Derom, Sinan I. Gülöksüz, Marc De Hert, Nele Jacobs, Claudia Menne-Lothmann, Bart P. F. Rutten, Evert Thiery, Jim van Os, Ruud van Winkel, Marieke Wichers, Inez Myin-Germeys

Only 18 of the 135 participants who endorsed feeling down within the last 12 months also reported experiencing suicidal ideation during this time. As discussed in S2, this rendered our original analysis plan unfeasible. For transparency, we nevertheless report the results of the planned analysis below.

## Statistical analysis

To investigate the relationship between daily positive future thinking and past-year suicidal ideation, we estimated mixed effects linear regression models with daily positive future thinking as the dependent variable, and past-year suicidal ideation as the independent variable. To investigate whether multilevel modelling was appropriate for these data, an unconditional model was estimated, which included only the outcome (future thinking) and the random intercept (participant ID). This model was statistically significant, indicating the multilevel modelling was appropriate. The associations between daily positive future thinking and suicidal ideation when taking into account positive and negative affect were investigated using mixed effects linear regression models. All models included age and sex as covariates. Given the extremely low number of individuals reporting suicidal ideation in the past-year, we do not report p values here or describe results in terms of statistical significance (apart from the unconditional model), as this will not provide useful information.

## Results

For model summaries see Table S3

There was a negative association between daily positive future thinking and past-year suicidal ideation, such that individuals reporting suicidal ideation also reported lower daily levels of positive future thinking. There was a positive association between daily future thinking and average positive affect from the previous day, with individuals reporting more average positive affect on the previous day also reporting higher levels of positive future thinking. There was a negative association between

daily positive future thinking and average negative affect from the previous day, such that lower levels of negative affect were associated with higher levels of positive future thinking.

*Table S3*
*Multilevel model summaries for original postregistered analysis*

| | Daily positive future thinking | | Daily positive future thinking | | Daily positive future thinking | | Daily positive future thinking | |
|---|---|---|---|---|---|---|---|---|
| *Predictors* | *Estimates* | *CI* | *Estimates* | *CI* | *Estimates* | *CI* | *Estimates* | *CI* |
| (Intercept) | 4.93 | 4.86 – 5.00 | 4.76 | 3.47 – 6.05 | 5.10 | 3.70 – 6.49 | 5.00 | 3.62 – 6.38 |
| Past year suicidal ideation | | | -0.47 | -0.98 – 0.04 | -0.37 | -0.94 – 0.20 | -0.36 | -0.92 – 0.20 |
| Sex | | | -0.18 | -0.53 – 0.17 | 0.01 | -0.38 – 0.39 | 0.01 | -0.37 – 0.39 |
| Age | | | 0.02 | -0.05 – 0.08 | -0.02 | -0.09 – 0.05 | -0.01 | -0.08 – 0.06 |
| Average positive affect from previous day | | | | | 0.36 | 0.08 – 0.64 | | |
| Average negative affect from previous day | | | | | | | -0.36 | -0.75 – 0.04 |
| **Random Effects** | | | | | | | | |
| $\sigma^2$ | 1.50 | | 1.87 | | 1.58 | | 1.57 | |
| $\tau_{00}$ | 0.64 subjid | | 0.50 subjid | | 0.62 subjid | | 0.59 subjid | |
| $\tau_{11}$ | | | | | 0.33 subjid.cent_day_pa_lag | | 0.77 subjid.cent_day_na_lag | |
| $\rho_{01}$ | | | | | -0.16 subjid | | -0.12 subjid | |
| ICC | 0.30 | | 0.21 | | 0.30 | | 0.31 | |
| N | 737 subjid | | 135 subjid | | 132 subjid | | 132 subjid | |
| Observations | 3474 | | 599 | | 464 | | 463 | |
| Marginal $R^2$ / Conditional $R^2$ | 0.000 / 0.300 | | 0.015 / 0.222 | | 0.018 / 0.317 | | 0.013 / 0.317 | |
| AIC | 12079.709 | | 2195.228 | | 1681.485 | | 1677.888 | |
